# Sacral chordoma in an adult showing an aggressive clinical course: A case report

**DOI:** 10.3892/ol.2014.1892

**Published:** 2014-02-19

**Authors:** KOJI ENDO, HIDEKI YAMASHITA, HIDEKI NAGASHIMA, RYOTA TESHIMA

**Affiliations:** Department of Orthopedic Surgery, Faculty of Medicine, Tottori University, Yonago, Tottori 683-8504, Japan

**Keywords:** chordoma, sacrum, Ki-67, aggressive

## Abstract

The current report presents a case of a 78-year-old male with sacral chordoma, showing an aggressive clinical course. The patient underwent sacral resection, however, nine months later, multiple metastases were detected by magnetic resonance imaging. The metastases progressed rapidly and 15 months following surgery the patient succumbed to respiratory dysfunction. An autopsy revealed multiple metastases of the lung, liver, heart, kidneys and vertebrae. Pathologically, the tumors did not show proliferation of anaplastic cells or dedifferentiation; however, the metastatic tumor cells were smaller than the primary tumor cells. The Ki-67 labeling indices were <5% in all of the patient’s tumors, therefore, the capacity for cellular proliferation of the tumors was considered to be low. Chordoma in adults are generally slow-growing tumors and are associated with a relatively prolonged course and frequent local recurrences. Therefore, it must be recognized that chordoma may grow rapidly and show an aggressive clinical course, even when the Ki-67 labeling index is low.

## Introduction

Chordoma is a rare tumor that constitutes only 3–4% of all primary bone tumors (1) and is considered to arise from the remnants of a primitive notochord. Chordoma arise from the sacrococcygeal region, base of the skull and vertebral column, with a common onset age of 60 years (1,2). The tumor usually develops in adults and grows slowly, however, repeated local recurrences occasionally occur (3,4).

Previous studies have demonstrated that chordoma in children has a greater tendency to metastasize than those in adults (5,6). Furthermore, the clinical course of chordoma in children is different compared with that in adults. The reason for this difference is that chordoma in children frequently exhibits an atypical histology with a diffuse and solid growth pattern, an increased degree of nuclear atypia and high mitotic activity. In adult cases, the clinical course generally shows indolent progression (1,2,7). Certain studies have reported that the local recurrence rates in adult cases were 33–71% (8,9) and Hulen *et al* (10) identified that following a surgical procedure the mean time of first recurrence was 29 months (range, 12–66 months) and the mean time of metastases was 50 months (range, 16–122 months). In addition, previous studies demonstrated that the five- and 10-year overall survival rates of chordoma patients were 74–83 and 50–56%, respectively (8,11,12).

The current study encountered a rare case of sacral chordoma in an adult who exhibited multiple metastases nine months following surgery and subsequently succumbed to the disease six months later. This case is presented along with the autopsy observations, which demonstrated an aggressive clinical course, although the tumor did not result in a sarcomatoid change. The family of the patient provided written informed consent.

## Case report

**Figure 1:**
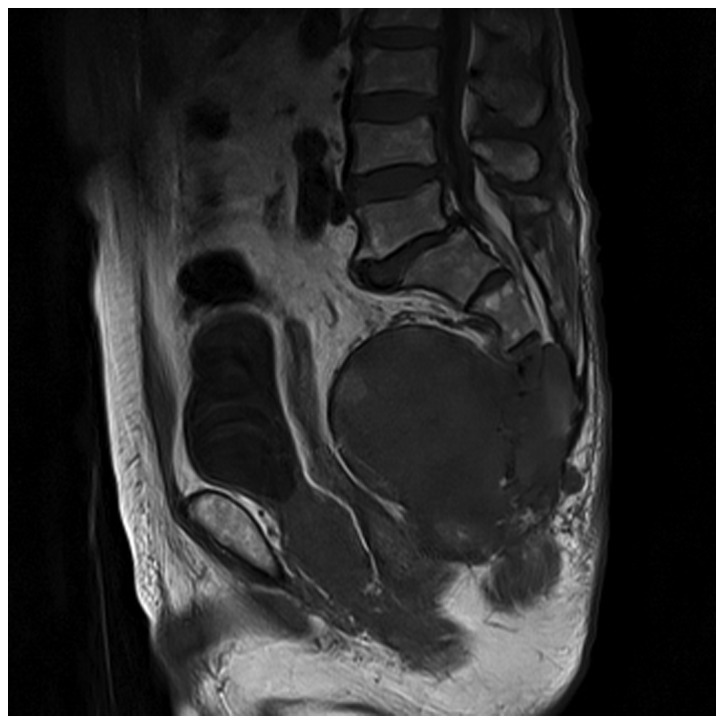
Magnetic resonance image demonstrated a large sacral tumor that spread into the soft tissue and compressed the rectum.

A 78-year-old male was admitted to an orthopedic clinic with a slow-growing, hard mass of the sacral region, which had developed over approximately two years, as well as chronic constipation. A large sacral mass was detected by magnetic resonance imaging (MRI) and the patient was referred to the Department of Orthopedic Surgery, Faculty of Medicine, Tottori University (Yonago, Japan) for evaluation and treatment. A physical examination revealed a diphasic elastic hard mass, measuring 8 × 3 cm in diameter with a smooth surface, in the sacral and gluteal regions. The mass was fixed to the sacrum and not adhered to the skin. The results of the neurological assessment were normal, with the exception of bowel dysfunction. Radiographs showed an osteolytic lesion in the sacrum, and MRI revealed a large tumor and compression of the rectum (Fig. 1). An open biopsy was conducted and histology of the specimen confirmed the diagnosis of a chordoma. Sacral amputation at S2 was performed along with resection of the soft component of the tumor. Adhesion to the presacral membrane was not prominent. The surgical margin was minor, although microscopically the margin was identified as R0. Adjuvant radiotherapy was initiated two months postoperatively due to an infection that was associated with catheter use and surgical wound dehiscence. An infection of the sacral region was revealed following radiotherapy (60 Gy), which was treated via curettage and administration of an antimicrobial agent. The patient was subsequently discharged.

**Figure 2:**
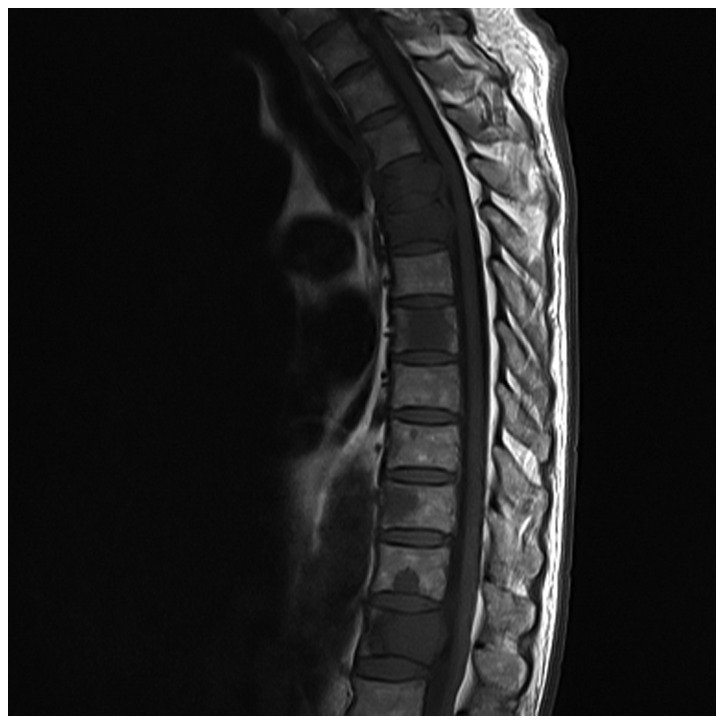
Magnetic resonance image of the vertebrae nine months following surgery revealed multiple vertebral metastases; the metastatic lesion at the 4th thoracic vertebra had compressed the spinal cord.

Nine months postoperatively, the patient complained of back and left shoulder girdle pain. MRI revealed multiple low intensity areas in the thoracic spine (4th, 5th, 7th, 10th and 12th vertebrae), and the spinal canal narrowed at the 4th and 5th thoracic vertebrae (Fig. 2). A needle biopsy and percutaneous vertebroplasty were performed on these lesions and a pathological examination demonstrated that these lesions were metastases of chordoma. Thereafter, metastatic lesions of the spine rapidly increased in size and number. In addition, a recurrent tumor was detected in the sacral region. Two months later, computed tomography and MRI detected metastases to the liver, cervical spine and right scapula. Tetraplegia subsequently occurred and gradually advanced, and four months later the patient succumbed to respiratory dysfunction. A subsequent autopsy demonstrated multiple metastases to the liver, vertebrae, kidneys, heart, pancreas and cervical lymph nodes. Pathological observations revealed apparent tumor emboli of the lungs. The causes of mortality were, therefore, identified to be pulmonary tumor emboli and respiratory dysfunction resulting from congested lungs.

**Figure 3:**
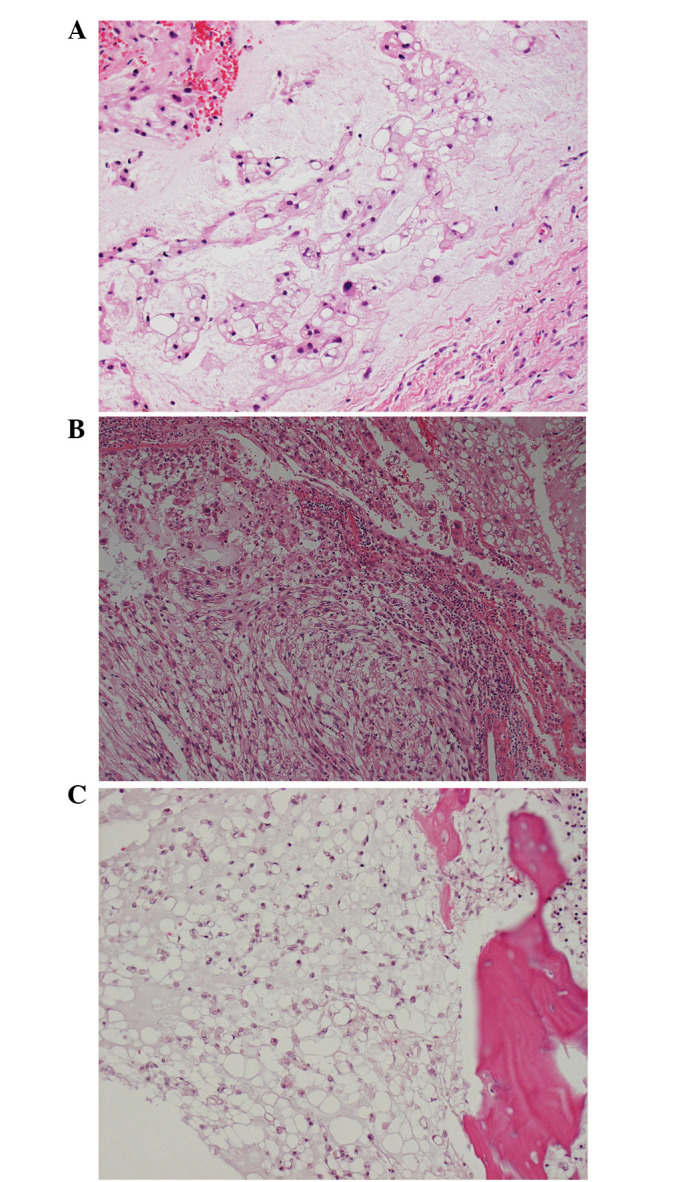
Histology of the tumors. (A) Physaliferous cells were demonstrated in a myxoid matrix of the primary tumor (hematoxylin and eosin [H&E] stain; magnification, ×200). (B) An area of atypical features was identified in the fibrous cells of the primary tumor. However, no apparent proliferation of anaplastic cells was identified. In addition, tumor cells did not demonstrate bizarre nuclei or sarcomatous features (H&E stain; magnification ×100). (C) The metastatic tumor cells were smaller than the cells of the primary tumor. These stellate cells were predominant in the metastatic lesions (H&E stain; magnification, ×200).

Histological examination of the primary tumor demonstrated that physaliferous cells were embedded in a myxoid matrix; spindle cells were also observed in other areas. The spindle cells did not exhibit nuclear atypia (Fig. 3A and B). In one of the metastatic tumors, the tumor cells were smaller than those in the primary region (Fig. 3C). These cells were termed stellate cells and no proliferation of anaplastic cells was identified in the primary or metastatic tumor.

**Figure 4:**
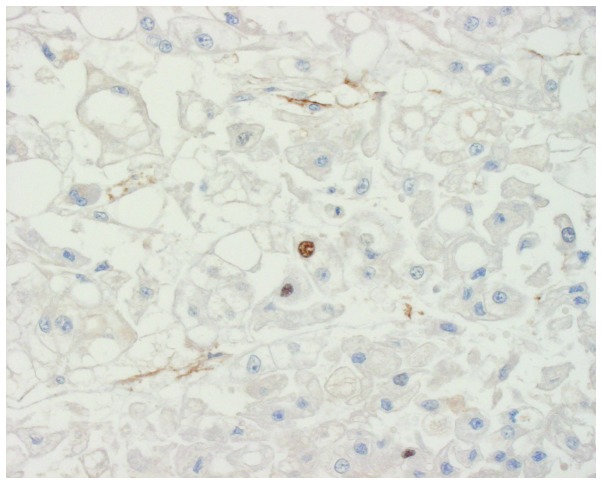
Immunohistochemistry (Ki-67 immunolabeling) demonstrated sparse staining for Ki-67 in the tumor cells of the primary tumor.

Upon immunohistochemical staining of the primary and metastatic tumors, the tumor cells were found to be positive for epithelial membrane antigen (EMA), cytokeratin and vimentin. However, the metastatic tumor cells were only moderately stained for vimentin and the Ki-67 labeling indices were <5% in the two tumors (Fig. 4). Therefore, the capacity for cellular proliferation of the tumors was considered to be low.

## Discussion

Chordomas are regarded as a low-grade malignancy, however, these lesions tend to recur locally and to metastasize to distant sites due to the specificity of their localization. Sites of chordoma involvement are the axial spine (sacral, 60%; spheno-occipital/nasal, 25%; cervical, 10%; and thoracolumbar, 5%) (13).

Sacrococcygeal chordomas in adults are generally considered to be slow-growing (2). However, previous studies of cases in infants described sacral chordomas with an aggressive clinical course (14,15). Shinmura *et al* (14) reported an autopsy case of a three-year-old male showing a tumor composed of ‘pink’ cells, which were hypothesized to reflect the earliest embryonic organs of the notochord, along with scattered physaliferous cells within a myxoid matrix. This case showed occasional mitotic figures with mild nuclear atypia. The tumor was not termed dedifferentiated chordoma, but atypical chordoma, since the tumor cells were positive for epithelial markers, such as cytokeratin and EMA, and did not demonstrate bizarre nuclei or sarcomatous features. In adiditon, Iwasa *et al* (15) reported two atypical chordomas in infancy and described that the tumors did not show proliferation of anaplastic cells or features of dedifferentiation. Adult cases of aggressive sacrococcygeal chordomas have previously been reported; these cases showed dedifferentiation to the fibrosarcoma, osteosarcoma and malignant fibrous histiocytoma (3,4,16). The present case did not demonstrate such findings, although the tumor cells exhibited mild nuclear atypia, for example, the metastatic cells were smaller than the tumor cells from the first biopsy. These stellate cells are responsible for tumor progression and it is hypothesized that physaliferous cells are degenerated stellate cells. If stellate cells are predominant in a tumor, this indicates an aggressive clinical behavior (17,18). The present tumor did not show any sarcomatoid features and the tumor cells were positive for epithelial markers; therefore, the tumor was diagnosed as comprising of features of conventional and atypical chordoma.

Ki-67 protein is a cellular marker of proliferation (19) and the fraction of Ki-67-positive tumor cells (the Ki-67 labeling index) often correlates with the clinical course of cancer. Previously, Holton *et al* (20) and Bergh *et al* (21) reported that the presence of mitotic figures and/or a Ki-67 labeling index >5–6% were associated with faster growing tumors and earlier metastases. In the current study, the Ki-67 labeling indices in the resected and recurrent tumors were <5%. Therefore, these observations did not explain the aggressive clinical behavior of this case.

Previously, Klingler *et al* (22) investigated microsatellite instability in sacral chordoma. The study demonstrated that a patient, who manifested no microsatellite instability, but a loss of heterozygosity (LOH) at *9p* and *18q*, exhibited an aggressive clinical cancer course, presenting with lymph node metastasis and succumbing to widespread metastatic disease. In addition, Horbinski *et al* (23) indicated that chordoma with *9p* LOH and/or *9p21* homozygous deletion may deomonstrate a risk for a more aggressive clinical course and shorter survival. This observation was of interest to the present study, however, our patient was not investigated for chromosomal anomalies. It is possible that this chromosomal anomaly was present and should have been investigated during the open biopsy.

The effects of adjuvant therapies, such as chemotherapy and radiation therapy, for chordoma are not as apparent as the response to surgery. Shinmura *et al* (14) and Iwasa *et al* (15) indicated that chemotherapy did not appear to benefit the control of recurrent and metastatic tumors in the cases of infants; furthermore, radiation therapy was not identified to be effective. Conversely, York *et al* (24) demonstrated that conventional radiation therapy extended the disease-free interval for patients that received subtotal resection. The current case showed low proliferative activity, however, the progression of the recurrent or metastatic tumor was not controlled by radiation therapy. Therefore, the complete resection of chordoma was considered to be the most important approach in the present study, as has been indicated in previous reports (24,25).

In conclusion, orthopedic surgeons must be aware that sacral chordoma may become aggressive, even in adults. In addition, due to its potential prognostic relevance, chromosomal anomalies in chordoma must be investigated during open biopsies.
